# Charting pathways to climate change mitigation in a coupled socio-climate model

**DOI:** 10.1371/journal.pcbi.1007000

**Published:** 2019-06-06

**Authors:** Thomas M. Bury, Chris T. Bauch, Madhur Anand

**Affiliations:** 1 Department of Applied Mathematics, University of Waterloo, Waterloo, Ontario, Canada; 2 School of Environmental Sciences, University of Guelph, Guelph, Ontario, Canada; University of Tennessee Knoxville, UNITED STATES

## Abstract

Geophysical models of climate change are becoming increasingly sophisticated, yet less effort is devoted to modelling the human systems causing climate change and how the two systems are coupled. Here, we develop a simple socio-climate model by coupling an Earth system model to a social dynamics model. We treat social processes endogenously—emerging from rules governing how individuals learn socially and how social norms develop—as well as being influenced by climate change and mitigation costs. Our goal is to gain qualitative insights into scenarios of potential socio-climate dynamics and to illustrate how such models can generate new research questions. We find that the social learning rate is strongly influential, to the point that variation of its value within empirically plausible ranges changes the peak global temperature anomaly by more than 1°C. Conversely, social norms reinforce majority behaviour and therefore may not provide help when we most need it because they suppress the early spread of mitigative behaviour. Finally, exploring the model’s parameter space for mitigation cost and social learning suggests optimal intervention pathways for climate change mitigation. We find that prioritising an increase in social learning as a first step, followed by a reduction in mitigation costs provides the most efficient route to a reduced peak temperature anomaly. We conclude that socio-climate models should be included in the ensemble of models used to project climate change.

## Author summary

The importance of anthropogenic CO_2_ emissions on climate change trajectories is widely acknowledged. However, geophysical climate models rarely account for dynamic human behaviour, which determines the emissions trajectory, and is itself affected by the climate system. Here, using a coupled socio-climate model, we show how social processes can strongly alter climate trajectories and we suggest optimal intervention pathways based on the model projections. Steps to increase social learning surrounding climate change should initially be prioritised for maximum impact, making a subsequent reduction in mitigation costs more effective. Policymakers will benefit from a better understanding of how social and climate processes interact, which can be provided by socio-climate models.

## Introduction

According to many ancient myths, humans did not invent fire-making de novo but rather learned it from personalities like Prometheus and subsequently spread the practice amongst themselves. These stories reveal how ancient myth-makers already grasped the fundamental importance of social learning—the process whereby individuals learn new behaviours, values and opinions from others [1]. Social learning is no less relevant in the era of human-environment challenges [2–4]. The importance of social learning and social processes more generally in climate change mitigation and adaptation is well recognised [5–8]. Increasingly sophisticated geophysical climate models are helping us understand the impacts of anthropogenic greenhouse gas (GHG) emissions [9–11], and the importance of these models is hard to understate. However, climate projections depend strongly on the assumed trajectory of GHG emissions [12]. This trajectory is determined by human behaviour and yet climate models generally do not incorporate dynamic social processes relevant to GHG emissions. Rather, GHG emissions are assumed to follow some specified trajectory. These trajectories are constructed with socio-economic factors in mind, (see Representative Concentration Pathways [12] and Shared Socioeconomic Pathways [13] for instance), but are not coupled to climate dynamics and do not capture human responses to climate change in a mechanistic way.

Just as human behaviour influences climate trends, climate change in turn influences human behaviour concerning GHG emissions, including both climate change mitigation and adaptation [5, 6, 8, 14, 15]. Individuals in places with rising average temperatures are more likely to perceive climate change [15], and social effects are apparent when individuals take steps in response to such shifting perceptions [6, 8]. There is also an important distinction between social learning and social norms—socially accepted and widely practised modes of conduct [16]. Social norms are known to have a strong influence on human behaviour [17] including aspects relating to climate change [7, 16, 18] and therefore play an important role in determining emission trajectories [6]. Multiple studies show a tendency for individuals to conform to emerging norms in support of climate change mitigation [7, 16]. Moreover, it appears that individuals are often not consciously aware of the importance of social norms in their decision-making and instead falsely ascribe their decisions to other factors [18]. However, it is important to note that social norms do not automatically promote socially beneficial outcomes. They can equally well force conformity to a destructive norm such as political extremism [19]. This also happens in the context of climate change behaviour, where it has been found that individuals may also conform to a norm of non-mitigation, by adjusting their habits to match those of less environmentally friendly neighbours [18, 20].

Hence, Earth’s climate and human subsystems are part of a single coupled system where social dynamics play a vital role. Yet, models of Earth’s coupled climate-behaviour system remain essentially undeveloped. One such approach [21] couples a sophisticated climate model [11] to a model for individual behavioural change based on the theory of planned behaviour—a dominant paradigm in psychology [22]. The authors find that the sensitivity of global temperature change to human factors such as response to extreme events, social norms and perceived ability to adopt mitigative strategies is of a similar magnitude to its sensitivity to geophysical factors. They deduce that quantifying behavioural uncertainty and physical uncertainty in climate projections deserve equal attention. The model focuses on how individual psychology and behaviour are influenced by extreme weather events. Social effects are modelled phenomenologically (i.e., exogenously imposed): individuals do not learn behaviour or opinions from one another, and social norms are treated as a fixed effect that does not depend on the population’s current composition of attitudes.

Here, we treat social learning and social norms endogenously, by modelling their dynamics as they emerge from rules governing how individuals interact, learn and behave. Our first objective is to develop qualitative insights into how different aspects of the system—endogenous social processes, temperature trends, and mitigation costs—separately and together determine possible dynamics of the larger socio-climate system. Our second objective is to illustrate potential uses of coupled socio-climate models to chart social and economic policy pathways that mitigate climate change as quickly as possible. To meet these objectives, we sought to develop a model that (1) could capture a range of IPCC climate change scenarios, ranging from 4 degrees of warming by 2100 (RCP 8.5 scenario) to sub 2 degrees of warming (RCP 2.6 scenario), (2) was simple enough to analyse so that we could learn which mechanisms drive the predicted socio-climate dynamics, (3) was based on existing approaches for modelling social dynamics and climate dynamics, and (4) captured the salient features of social and climate systems. Given the model’s simplicity, it is primed for insights as to how social and climate processes interact, though limited in its predictive capacity due to the complexity of the socio-climate system. The development of more complex socio-climate models will be an important research avenue, once the mechanisms of socio-climate dynamics are better understood.

## Materials and methods

### Model

Geophysical models in the climate science literature span a wide range of different complexities depending on the associated research objective. Highly complex models are the state-of-the-art for weather and climate prediction [11, 23, 24], whereas simple models allow us to assess processes and feedbacks, thereby improving our intuition of climate system dynamics [25–29]. Likewise, the behavioural sciences have benefited from a variety of modelling approaches, that address the diverse set of social processes that take place on the individual and societal level [30]. Here, we use minimal models for both social and climate dynamics. Starting simple allows us to build intuition on the effect of socio-climate feedbacks that have yet been considered in the climate change literature. The social model is widespread and, despite its simplicity, captures the salient aspects of social dynamics [2, 30, 31]. Moreover, the simple Earth system model that we use [25] accurately follows the projections of the state-of-the-art CMIP5 models when forced with the IPCC emission scenarios (S1 Fig).

#### Behaviour dynamics

Individuals in our model are either ‘mitigators’ or ‘non-mitigators’ and they learn these behaviours from others at a specified social learning rate. They switch to the more attractive behaviour according to a utility function governed by the costs of climate change mitigation (such as the cost of installing solar panels or buying gas-electric hybrid vehicles), the costs imposed on non-mitigative behaviour (such as a carbon tax), the costs associated with the average global temperature anomaly, and the utility associated with social norms that reinforce the majority behaviour, whether it be mitigation or non-mitigation. Social norms strengthen as the majority behaviour becomes more prevalent, consistent with empirical studies [7, 18]. Formally, the utility of being a mitigator is taken as

$$\begin{array}{c} e_{M}=-\alpha +c\tilde{f}\left(T_{f}\right)+\delta x \end{array}$$ (1)

where *α* is the cost of adopting mitigative strategies, *c* is a proportionality constant that regulates the extent to which climate change costs influence incentive to mitigate, $\tilde{f}\left(T\right)$ is the cost associated with with a temperature anomaly of *T* degrees Celsius, *T*_*f*_ is a projected temperature anomaly (see below), *δ* is the strength of social norms and *x* is the proportion of mitigators in the population. The utility of being a non-mitigator is taken as

$$\begin{array}{c} e_{N}=-\gamma -\tilde{f}\left(T_{f}\right)+\delta \left(1-x\right), \end{array}$$ (2)

where *γ* is the cost of non-mitigative behaviour, representing for example a carbon tax. Note that the utility due to social norms here is instead weighted by the proportion of non-mitigators, 1 − *x*.

Social learning is implemented as follows. Each individual samples other members of the population at a fixed rate *κ*. If ‘individual A’ samples ‘individual B’ and B’s strategy has a higher payoff that of A, then A will switch to B’s strategy with a probability proportional to the difference in payoffs. Thus if there are *x* mitigators in the population, the rate at which a non-mitigator encounters a mitigator is *κx*. Should there be a utility gain in switching (*e*_*M*_ > *e*_*N*_), the non-mitigator will switch their strategy with a probability proportional to the payoff difference, *e*_*M*_ − *e*_*N*_. Since there are a total of 1 − *x* non-mitigators in the population, the total rate at which non-mitigators switch to being mitigators is

$$\begin{array}{c} r_{N\to M}=\kappa x\left(1-x\right)\;\text{max}\left\{e_{M}-e_{N},0\right\}. \end{array}$$ (3)

Using similar arguments, one can show

$$\begin{array}{c} r_{M\to N}=\kappa x\left(1-x\right)\;\text{max}\left\{e_{N}-e_{M},0\right\} \end{array}$$ (4)

and so the net rate of change in mitigators is

$$\begin{array}{c} \frac{dx}{dt}=r_{N\to M}-r_{M\to N}=\kappa x\left(1-x\right)\left(e_{M}-e_{N}\right), \end{array}$$ (5)

which has an equivalent form to the replicator equations of evolutionary game theory [32]. Writing the payoff functions explicitly gives

$$\begin{array}{c} \frac{dx}{dt}=\kappa x\left(1-x\right)[\gamma -\alpha +\left(c+1\right)\tilde{f}\left(T_{f}\right)+\delta \left(2x-1\right)], \end{array}$$ (6)

which can be reduced to

$$\begin{array}{c} \frac{dx}{dt}=\kappa x\left(1-x\right)[-\beta +f\left(T_{f}\right)+\delta \left(2x-1\right)], \end{array}$$ (7)

by introducing the new parameter *β* = *α* − *γ* (the net cost to mitigate) scaled function $f\left(T\right)=\left(c+1\right)\tilde{f}\left(T\right)$ (the net temperature associated gain to mitigate).

#### Temperature projection

Because long-term climate forecasts are known to influence individual decisions on whether to support mitigation [14], the utility function assumes that individuals base decisions on long-term extrapolations of recently experienced climate trends. The projected temperature takes the form

$$\begin{array}{c} T_{f}\left(t\right)=T\left(t\right)+(\frac{t_{f}}{t_{p}})\left(T\left(t\right)-T\left(t-t_{p}\right)\right) \end{array}$$ (8)

where *t*_*p*_ is the number of years back to extrapolate from and *t*_*f*_ is the number of years forward to extrapolate to.

#### Perceived costs associated with climate change

We assume that the costs associated with climate change have a sigmoidal relationship with the global temperature anomaly. This is motivated by the slow mitigative response to global warming over the past decade (S8–S10 Figs) and the anticipated non-linear alterations in the Earth system with increasing temperature [33]. Specifically

$$\begin{array}{c} \tilde{f}\left(T\right)=\frac{{\tilde{f}}_{\text{max}}}{1+e^{-\omega (T-T_{c})}}, \end{array}$$ (9)

where ${\tilde{f}}_{\text{max}}$ corresponds to a maximum cost, *ω* is the degree of nonlinearity of the sigmoid, and *T*_*c*_ is the critical temperature about which costs are most sensitive to change (S5 Fig). We combine *c* and ${\tilde{f}}_{\text{max}}$ into the single parameter

$$\begin{array}{c} f_{\text{max}}=\left(c+1\right){\tilde{f}}_{\text{max}}, \end{array}$$ (10)

which yields the functional form

$$\begin{array}{c} f\left(T\right)=\frac{f_{\text{max}}}{1+e^{-\omega (T-T_{c})}}, \end{array}$$ (11)

as used in (7).

#### Earth system model

We couple the social model to an Earth system model [25] with reduced ocean dynamics [34]. Dynamics for atmospheric CO_2_ is modified to include an anthropogenic emission term, dependent on the proportion of non-mitigators. Specifically

$$\begin{array}{c} \frac{dC_{\text{at}}}{dt}=\epsilon \left(t\right)\left(1-x\right)-P+R_{\text{veg}}+R_{\text{so}}-F_{\text{oc}} \end{array}$$ (12)

where *C*_at_ is the deviation in atmospheric CO_2_ from pre-industrial values, *ϵ*(*t*) is the baseline rate of CO_2_ emissions in the absence of mitigation, *P* is the rate of carbon uptake via photosynthesis, *R*_*veg*_ is the outward carbon flux via plant respiration, *R*_*so*_ is the outward carbon flux via soil respiration and *F*_*oc*_ is the net uptake of carbon by the oceans. Functional forms for the transfer of carbon via these processes are provided in S1 Text. Global surface temperature is assumed to evolve with the carbon cycle according to [25]

$$\begin{array}{c} c\frac{dT}{dt}=\left(F_{d}-\sigma T^{4}\right)a_{E} \end{array}$$ (13)

where *T* is the deviation in global surface temperature from pre-industrial values, *c* is the thermal capacity specific heat capacity of the Earth’s surface, *F*_*d*_ is the net downward flux of radiation absorbed at the planet’s surface (which depends on the opacity of CO_2_), *σ* is the Stefan-Boltzmann constant, and *a*_*E*_ is the Earth’s surface area. Full details are provided in S1 Text.

### Simulation

Over the period from 1800 to 2014, the socio-climate model is simulated with a fixed social component, forced with historical anthropogenic carbon emissions. Initial conditions for all climate variables are zero since they represent deviations from pre-industrial values. Social dynamics are initiated in 2014 with an initial proportion of mitigators *x*_0_ = 0.05. The ensuing dynamics of *ϵ*(*t*) follow an increasing but saturating trend corresponding to the world’s increasing but saturating population size and energy demands. Specifically

$$\begin{array}{c} \epsilon \left(t\right)=\{\begin{array}{cc} \text{linear}\;\text{interpolation}\;\text{of}\;\text{historical}\;\text{emissions} & t\leq 2014\\ \epsilon_{2014}+\frac{\left(t-2014\right)\epsilon_{\text{max}}}{t-2014+s} & t\geq 2014 \end{array} \end{array}$$ (14)

where *ϵ*_max_ is the saturating value, and *s* the half-saturation constant, of *ϵ*(*t*). This expression is shown graphically in S7 Fig. The system of (delay) ordinary differential equations is simulated using the NDSolve package in Wolfram Mathematica. Historical CO_2_ emissions were obtained from the CDIAC data repository [35].

### Parameters and sensitivity analysis

Baseline climate parameters are obtained from the original Earth system model [25] where they were fitted to obtain historical trends of temperature and carbon dynamics. Social parameters are more speculative and so are given wide upper and lower bounds. The relative cost of warming (*f*(*T*)) with respect to the net cost of mitigation (*β*) is chosen in accordance with the argument that the costs of preventative action will be far less than the cost implied otherwise by global warming [36]. For sensitivity analyses we draw parameters from triangular distributions that peak at baseline values and extend to upper and lower bounds (S2 Table). Parameters are kept fixed preceding 2014 to retain historical trends in the simulations.

## Results

**Fig 1:**
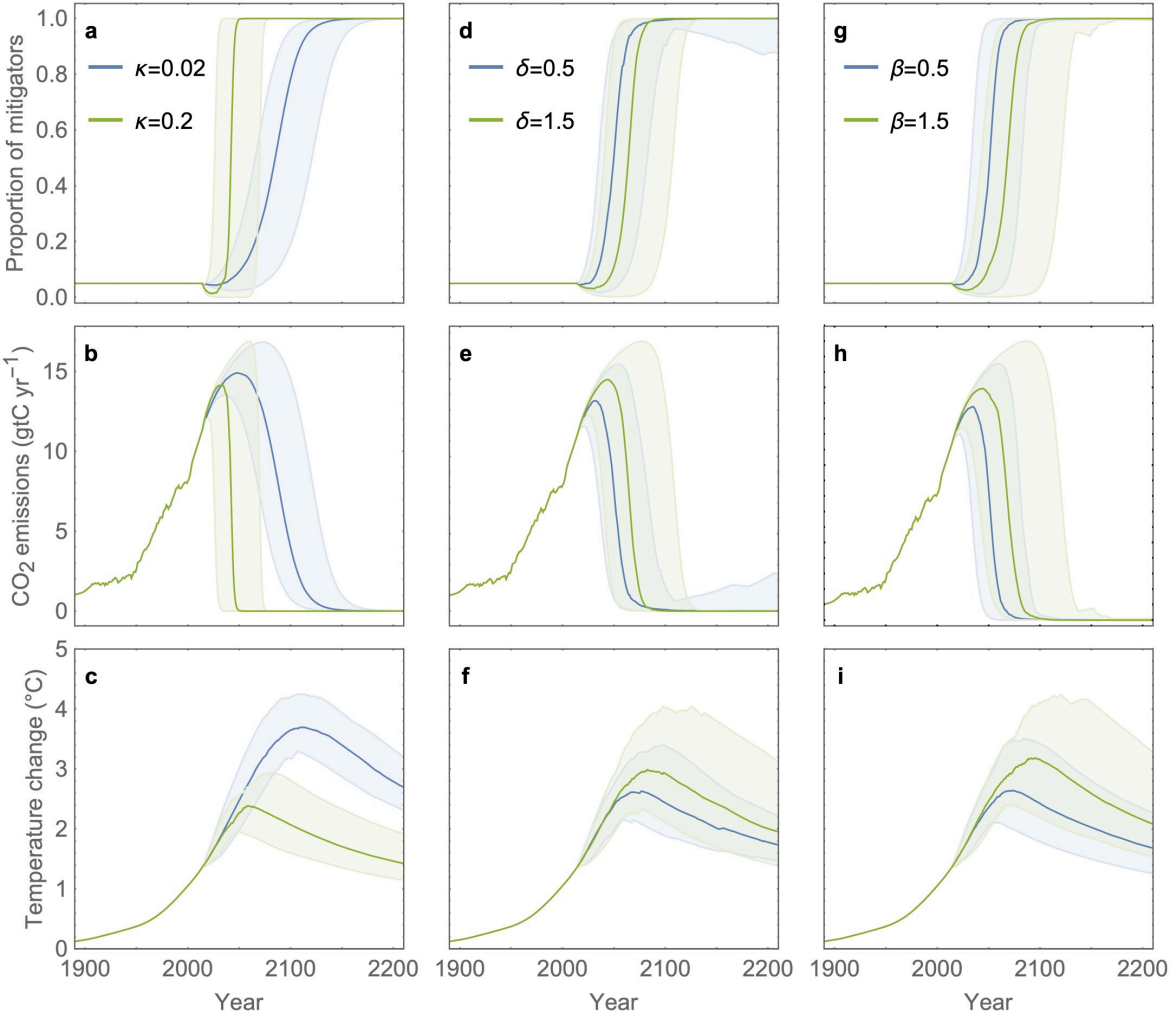
Endogenous social dynamics influence climate trajectories. Shown are ensembles of model simulations comparing two parameter values for the social learning rate (*κ*) (a-c), strength of social norms (*δ*) (d-f), and costs associated with mitigation (*β*) (g-i). All other parameters are drawn from triangular probability distributions with defined upper, lower and baseline values (S2 Table). Displayed are median trajectories with 95% confidence intervals from an ensemble of 100 realisations. Additional details in Methods.

The model demonstrates how the social learning rate can strongly determine temperature trends. We first consider a null hypothesis where adaptive behaviour is removed from the model by forcing the proportion of mitigators in the population to remain constant. In this case of fixed behaviour, emissions saturate and the temperature anomaly increases indefinitely (S2 Fig). However, once social learning is added and the proportion of mitigators is allowed to evolve dynamically as in our baseline model, the predicted average global temperature anomaly can peak anywhere from 2.2°C, near the Intergovernmental Panel on Climate Change (IPCC) limit [37] (in the case of very rapid social learning) to 3.5°C (in the more realistic case where social learning unfolds on a generational timescale) (Fig 1a–1c). Whether people discuss climate change more or less often can therefore strongly influence temperature trends. Because we model social norms as something that tends to reinforce majority behaviour and attitudes—whatever they might be—one might think that social norms act as a double-edged sword. In fact, they operate more like an unhelpful scimitar, as illustrated by comparing cases of low and high strength of social norms. Because the population starts off from a state of largely non-mitigating behaviour, increasing the strength of social norms suppresses the spread of mitigating behaviour for decades by entrenching non-mitigation as a norm, even when rising temperatures strongly justify an immediate shift (Fig 1d–1f). (This model dynamic echoes not only current climate norms reinforcing non-mitigation [20] but also past social shifts occurring on decadal timescales, such as evolving social norms about when and where smoking is acceptable.) However, when mitigating behaviour eventually does become widespread, a higher strength of social norms does not significantly accelerate its spread. Rather, the two curves for cases of high and low social norm strength simply move in parallel to one another because by this time, the utility function that determines behaviour change is dominated by the large temperature anomaly (Fig 1d). In this parameter regime, social norms generate a perverse asymmetry, in contrast to findings from other socio-climate models that assume social norms can only support climate change mitigation [20].

The model also shows how a reduction in net mitigation cost can significantly accelerate the onset of social change. For instance, a 67% reduction in the mitigation cost increases the percentage of mitigators by 2060 from 10% to 90% (Fig 1g–1i). Therefore, policies that reduce the cost of mitigation (through e.g. subsidies, tax cuts) will benefit from the accelerating effects of social learning and must be timed correctly.

**Fig 2:**
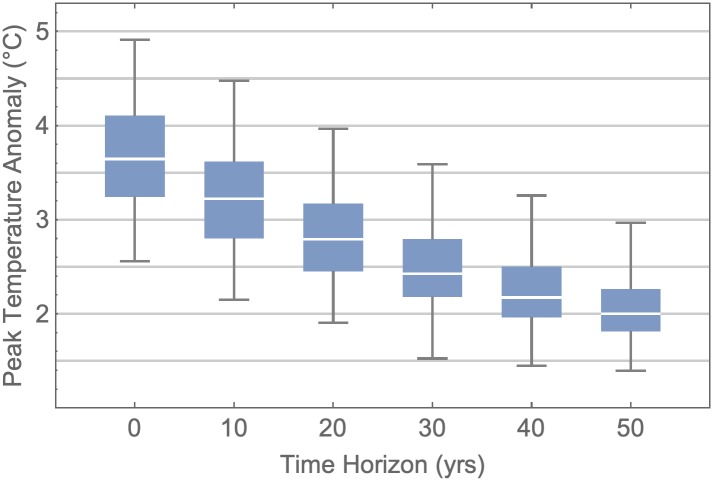
Peak temperature anomaly strongly depends on individual time horizon. Box-and-whisker plot shows the peak temperature anomaly measured over 100 realisations for fixed time horizons (*t*_*f*_) and all other parameters drawn from triangular probability distributions with defined upper, lower and baseline values (S2 Table). Boxes span the interquartile range, whiskers span the entire set of realisations, and box dividers mark the median. Additional details in Methods.

Our baseline model assumes that individuals’ perceived cost of climate change impacts depends on a linear extrapolation of the recent temperature anomaly over the previous ten years (Methods). If individuals instead base their decisions only on the current temperature anomaly, the simulated global temperature anomaly lies well above the 2°C target set by the IPCC, and exhibits wide variation in sensitivity analysis (Fig 2). This contrasts with our baseline model where the population movement towards mitigative strategies ignites earlier, significantly reducing the global temperature anomaly. This predicted dynamic stems from the multi-decadal lag between GHG emissions and the consequent global temperature rise [38].

**Fig 3:**
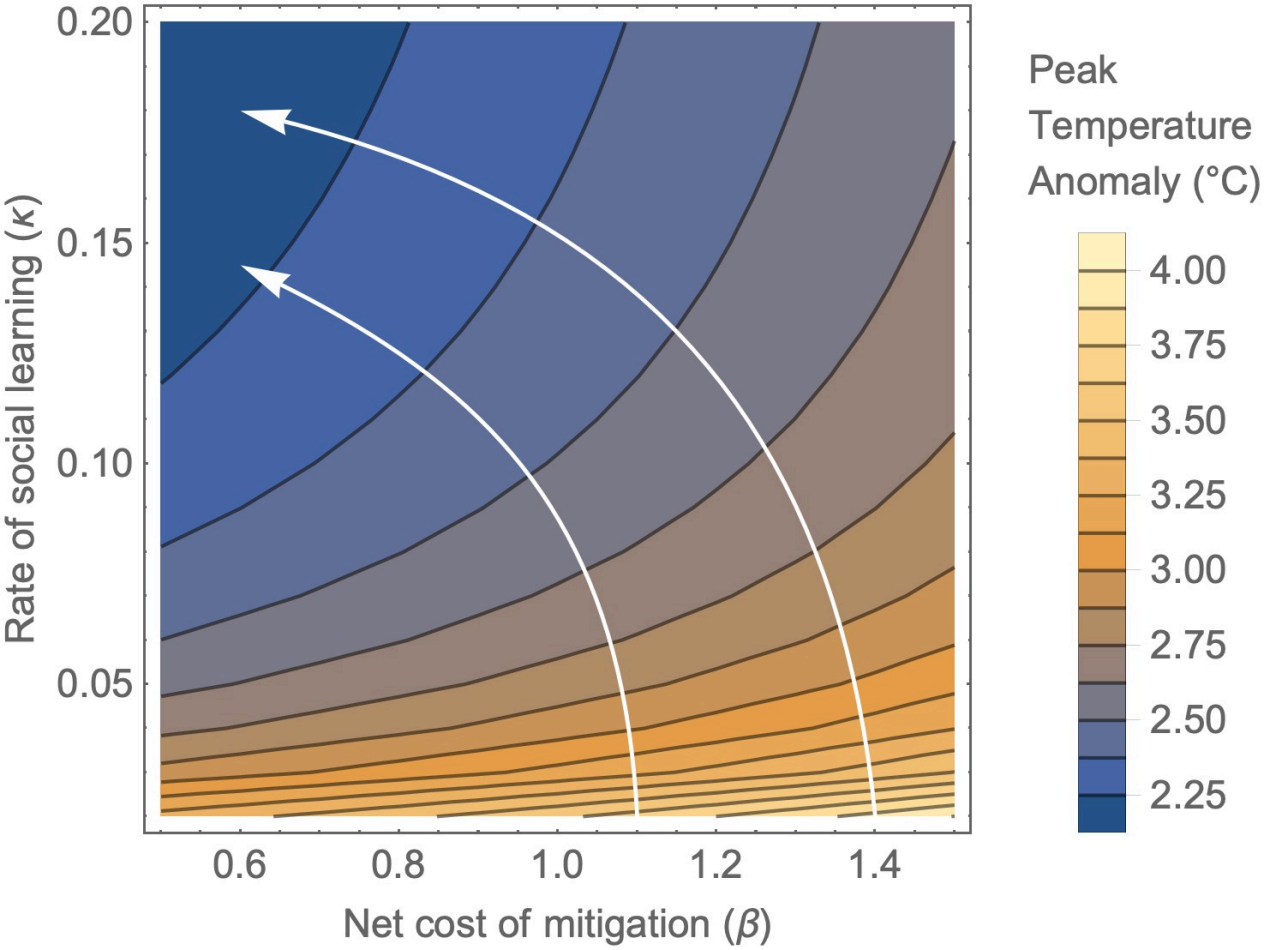
Optimal pathways to mitigation via increasing social learning and reducing mitigation costs. Contour plot showing peak temperature anomaly at specified values of the net cost of mitigation (*β*) and rate of social learning (*κ*). Arrows indicate the direction of steepest descent—the most efficient combination of the two measures to reduce temperature anomalies. All other parameters are fixed at baseline values (S2 Table). Additional details in Methods.

Our model predicts medium-term GHG emission trajectories (Fig 1b, 1e and 1h) that are qualitatively similar to those often assumed under various future emissions scenarios. This raises the question of how such models can be useful. The socio-climate model enables us to explore how socio-climate dynamics might respond to changes that are under the control of policymakers. For instance, it is possible to compare social and economic policy interventions by considering the effects of simultaneous parameter changes, instead of one at a time. This enables us to chart out the quickest pathways from highest to lowest temperature anomalies. The relative merits of increasing the social learning rate vs. reducing the net cost of mitigation are illustrated with a contour plot where the contours represent peak temperature anomaly as a function of the two parameters (Fig 3). Increasing the social learning rate (e.g. through media coverage and public fora devoted to climate change) is particularly effective when social learning is slow, but has saturating benefits, as indicated by the increasing vertical spacing of contour lines for at higher learning rates. In contrast, reducing the net mitigation cost (e.g. through tax breaks) drives a more linear response in peak temperature anomaly. Crucially, it should be noted that both a reduction of net mitigation cost and an increase in the social learning rate are required to achieve the IPCC target. The arrows in Fig 3 show the ‘path of steepest descent’—the most efficient combination of the two measures. Starting from a situation of high projected temperature anomalies, the model predicts that increasing the social learning rate should first be prioritised, followed by a reduction in net mitigation cost once the benefits of social learning begin to saturate. This approach gets us to the region of parameter space corresponding to the IPCC target faster than alternative trajectories.

**Fig 4:**
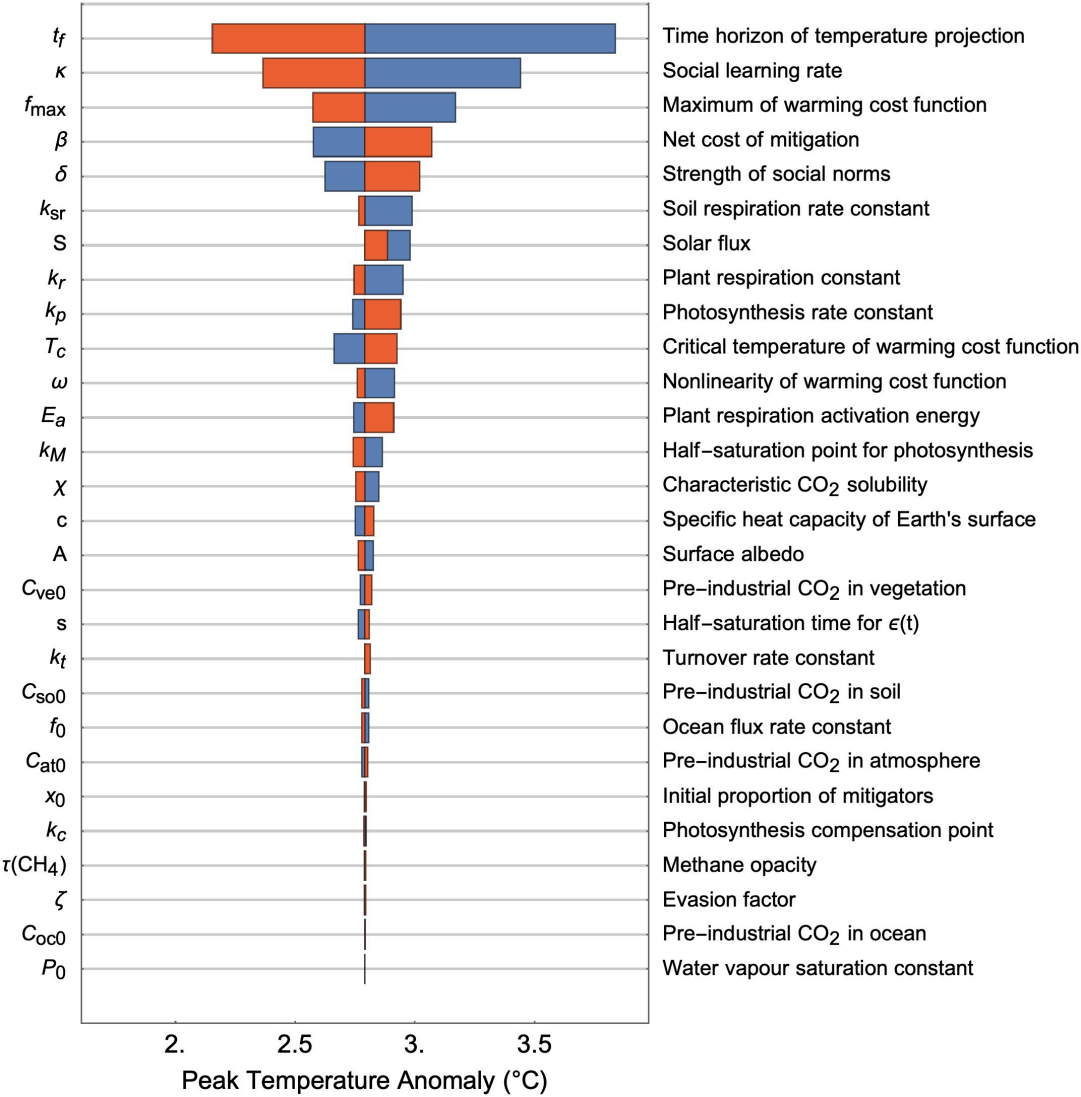
Peak temperature anomaly is sensitive to both social and climate parameters. Tornado plot showing the deviation in peak temperature anomaly when parameters are varied individually. A red (blue) bar indicates the deviation when the specified parameter adopts its upper (lower) bound, while all other parameters are fixed at baseline values (S2 Table). Parameters ordered by relative impact from top to bottom. Additional details in Methods.

A sensitivity analysis reveals the relative influence of each parameter on the peak temperature anomaly (Fig 4). The time horizon of individuals’ temperature projection, social learning rate and costs of mitigation are major factors, all of which may be influenced by appropriate intervention. The importance of social parameter uncertainties in determining climate predictions indicated by our model has also been predicted by other socio-climate models [21]. Interestingly, the system is relatively insensitive to the initial proportion of mitigators, suggesting that the mediation of social processes, as opposed to the current social state, is key to guiding the socio-climate system to a trajectory of reduced emissions. Sensitivity analyses such as these can help investigators determine priorities for data collection: the parameters exhibiting the greatest influence on predictions should be targeted for data collection so we can best reduce model uncertainty.

A striking feature revealed by the sensitivity analysis is the asymmetry in many of the parameter dependencies. Consider the three parameters with highest impact on the peak temperature anomaly (concerning forecast horizon, learning rate and global warming costs). A decrease in these parameters is more detrimental than an increase is beneficial. For example, a forecast horizon 10 years above baseline value results in a 0.6 degree decrease in peak temperature anomaly, whereas a forecast horizon 10 years below baseline value results in a 1 degree increase. This imbalance is a manifestation of the nonlinear interactions between and within each of the social and climate system.

The sensitivity analysis also reveals non-monotonic relationships between the peak temperature anomaly and the parameters. For example, both an increase and a decrease in solar flux results in a higher peak temperature anomaly. Interestingly, this is not the case if the climate subsystem is considered in isolation. For a fixed emissions scenario, a higher (lower) solar flux will always result in a higher (lower) peak temperature anomaly, since the solar flux is proportional to the net downward radiation absorbed by the planet’s surface. The coupling to social dynamics fundamentally alters this relationship. In the socio-climate system, a reduced solar flux results in a slower increase in surface temperature. As a consequence, individuals are less incentivised to mitigate, causing the social system to maintain a regime of non-mitigative behaviour. The accompanying high rate of CO_2_ emissions quickly overcompensates for the reduced solar flux, yielding a higher peak temperature anomaly. Thus seemingly useful interventions to the physical system can actually end up doing more harm than good when there is strong coupling to a social system, as is the case for global warming.

## Discussion

This study has shown how social processes can influence climate dynamics, according to one possible way of modelling social dynamics and norms. However, other frameworks for modelling human behaviour could yield different predictions. For instance, the socio-climate model of Ref. [21] does not include social learning. Individuals respond directly to changes in the climate, and not through interactions with one another. As a consequence, the rate at which individuals adopt mitigative strategies only varies with the current climate situation, and not with current population consensus. Mitigation efforts can therefore be expected to closely follow the severity of climate change in the model. In our model, social learning manifests as a feedback within the social system, resulting in qualitatively different socio-climate trajectories. Mitigative behaviour is initially suppressed–even as temperatures rise to levels that should incentivise mitigation–due to low numbers of mitigating individuals and therefore little turnover of behaviour in the population. However, social learning creates a positive feedback loop once there is a net positive utility to mitigate, and so as the numbers of mitigators increases, so too does the rate at which non-mitigators switch to being mitigators. This results in a sharp non-linear increase in mitigators, as a combined outcome of both the social and the climate system dynamics. We note that, all else being equal, adding social learning to a model has the effect of slowing down behaviour change in the human population (since a process takes time, by definition), and therefore the mitigation response of human populations.

Conversely, the case of very rapid social learning recovers a ‘best response’ model similar to those assumed in classical economics, where individuals immediately adopt the highest payoff strategy without learning the behaviour from others. Whether or not this assumption can approximate behaviour in real human populations hinges upon how fast social learning occurs—individuals would need to sample others rapidly enough to enable complete population behaviour change within 5 years for this approximation to work in our model, which seems implausible (Fig 1a–1c).

In a different vein, we assumed a homogeneous population with respect to mixing and individual utilities. The model’s social dynamics capture interactions at the individual level, though there are many different scales of social organisation that the model does not consider, from families/neighbourhoods to cities/states and up to interacting countries. Future models could include this more hierarchical social structure. Similarly, these models could include different types of individual with correspondingly different utilities. For instance, the model could include industrial corporations with utilities biased toward shareholder profit, and social institutions (such as laws, taxes, the education system) that reflect the current governmental stance. Social learning may also take on different forms due to diverse individual psychologies and values [39–41]. Such heterogeneities are known to affect the dynamics of a wide variety of systems [42] and can prevent population consensus by permitting development of echo chambers [43]. Our model also makes the simplifying assumption that individuals base their temperature projection on linear extrapolation of past temperatures. This could be generalised to a non-linear extrapolation to reflect an individual’s perception of ‘accelerating’ change. Extending socio-climate models to include these finer details should prove valuable in further investigations.

Climate change is a manifestation of coupled human-environment dynamics and therefore we should start coupling climate models to social models [5, 44]. Our simple coupled socio-climate model shows that the rate at which individuals learn socially strongly influences the peak global temperature anomaly, to the point that variation of this parameter within plausible ranges changes the peak temperature anomaly by more than 1°C. Therefore, it matters whether social processes cause slow or fast uptake of climate change mitigation measures. We found that social norms may not provide help when we most need it, although this finding could be nuanced by adding social heterogeneity. Finally, we illustrated how exploring the parameter space of socio-climate models suggests optimal paths for mitigating climate change. A more sophisticated policy impact assessment model based on a coupled socio-climate approach could therefore be useful to decision-makers facing a mandate to reduce GHG emissions with a fixed budget. In summary, it is essential for climate change research to account for dynamic social processes in order to generate accurate predictions of future climate trends, and the paradigm of coupled socio-climate modelling could help us address this challenge.

## Supporting information

S1 Text Formulation of socio-climate model. Includes full description and derivation of the socio-climate model and its parameters.
(PDF)

S1 Table Labels for state variables and dynamic processes in the socio-climate model. (PDF)

S2 Table Definitions and values for the parameters in the socio-climate model. (PDF)

S1 Fig Comparing temperature projections from the simple Earth system model with those of more complex climate models. **a**. Ensemble of simulations from the Coupled Model Intercomparison Project Phase 5 (CMIP5) using the Representative Concentration Pathways (RCPs) as emission scenarios (figure from the IPCC Fifth Assessment Report [45]). Displayed are 95% confidence intervals based on annual means. Numbers and their colour denote the number of models used for each RCP scenario. **b**. Ensemble of simulations from the simple Earth system model that we use in our socio-climate model under the same RCP emission scenarios. Parameters are drawn from triangular distributions with upper and lower bounds given in S2 Table.
(TIFF)

S2 Fig Climate trends with and without adaptation to climate change. Removing adaptive behaviour from the model (by forcing the proportion of mitigators to remain at a constant, low value) results in saturating emissions and temperature increasing indefinitely (at least over the next two centuries). This is akin to the RCP8.5 scenario in the latest IPCC report (trajectory shown in Fig 1). Simulations above use parameter values drawn from triangular distributions with upper and lower bounds given in S2 Table.
(TIFF)

S3 Fig Worst-case scenario of the coupled socio-climate model. Setting the social parameters to their bound that most favour non-mitigative behaviour, we get no spread of mitigative behaviour within the considered time-frame. This causes the temperature to increase in a manner similar to the RCP 8.5 scenario (S1 Fig). Fixed parameter values are *κ* = 0.02, *β* = 1.5, *δ* = 1.5, *f*_max_ = 4, *x*_0_ = 0.01. All other parameter values are drawn from triangular distributions with upper and lower bounds given in S2 Table.
(TIFF)

S4 Fig Best-case scenario of the coupled socio-climate model. Setting the social parameters to their bound that least favour non-mitigative behaviour, we get very early spread of mitigative behaviour. This causes the temperature to evolve in a manner most similar to the RCP 2.6 scenario where temperature change stays below 2 degrees Celsius (S1 Fig). Fixed parameter values are *κ* = 0.2, *β* = 0.5, *δ* = 0.5, *f*_max_ = 6, *t*_*f*_ = 50. All other parameter values are drawn from triangular distributions with upper and lower bounds given in S2 Table.
(TIFF)

S5 Fig Functional form for perceived costs associated with climate change. The incentive of individuals to mitigate is in part based on their perceived costs of climate change *f* at some projected temperature *T*. We adopt a sigmoidal response curve for *f*(*T*) with variable curvature *ω* and horizontal shift *T*_*c*_ (the explicit form of *f*(*T*) is provided in Methods). This form captures the expected non-linear increase in climate change impacts (cost) as temperature increases.
(TIFF)

S6 Fig Estimated historical CO_2_ emissions. Data from the CDIAC on carbon emissions due to fossil-fuel burning and land-use changes, during the years 1800-2014. Land-use data is only available from 1850-2005 and so we linearly extrapolate (dashed line) to match the range of the fossil-fuel data. The sum of the emission trajectories is used to drive the model up to the year 2014, from which point the behavioural component of the socio-climate model is initiated.
(TIFF)

S7 Fig Emissions in the absence of behavioural change. In the socio-climate model, the factor *ϵ*(*t*) corresponds to the global CO_2_ emissions should there be no change in human behaviour. Preceding 2014, *ϵ*(*t*) takes the historical emission trajectory (solid line) as human behaviour is not modelled here. Post 2014, *ϵ*(*t*) follows a saturating function (dashed line) to capture the saturation of global population size and energy needs. Details of this functional form can be found in the Methods section.
(TIFF)

S8 Fig Comparing per capita CO_2_ emissions with global temperature changes. Temperature anomaly (solid line) above the 1850-1900 baseline value and CO_2_ emissions per capita (dashed line) are shown for the 1850-2014. Data was obtained from the CDIAC data repository [35]. It is clear that despite exceeding a 1 degree temperature anomaly, the global emissions per capita of CO_2_ show no obvious signs of decreasing. The current temperature anomaly is not yet high enough to spark a global decrease in emissions.
(TIFF)

S9 Fig CO_2_ emissions by country. Total industrial CO2 emissions from the ten countries with the greatest cumulative output. Data was obtained from the CDIAC data repository [35]. Note the termination of the green curve marks the dissolution of the Soviet Union and the beginning of the yellow curve marks the formation of the Russian Federation.
(TIFF)

S10 Fig CO_2_ emissions per capita by country. Total industrial CO_2_ emissions per capita from the ten countries with the greatest cumulative output. Data was obtained from the CDIAC data repository [35]. From 1950-1980 we observe strong increases in emissions per capita among most of these countries as is seen globally in S8 Fig. Many of the countries peak in 1980 and follow slight downwards trends demonstrating a degree of country-level movement towards mitigation. Globally, this is not the case, however.
(TIFF)
