# Supplementary material for: Charting pathways to climate change mitigation in a coupled socio-climate model
Source: PLoS Comput Biol. 2019 Jun 6;15(6):e1007000. doi: 10.1371/journal.pcbi.1007000 (PMC6553685; doi:10.1371/journal.pcbi.1007000)
Supplement: S1 Text — Includes full description and derivation of the socio-climate model and its parameters. (PDF) [file pcbi.1007000.s001.pdf]

S1 Text: Formulation of socio-climate model

Thomas Bury[1,2,*], Chris T. Bauch[1], and Madhur Anand[2]

[1]Department of Applied Mathematics, University of Waterloo, Waterloo, ON N2L 3G1, Canada
[2]School of Environmental Sciences, University of Guelph, Guelph, ON N1G 2W1, Canada.

*email: tbury@uwaterloo.ca

1 Model components

We couple an Earth system model (ESM) [4] with reduced ocean dynamics [6], to a dynamic model for social behavior [1, 2, 3]. The full socio-climate model reads

$$\frac{dx}{dt} = \kappa x(1-x)(-\beta + f(T_f) + \delta(2x-1)), \tag{1}$$
$$\frac{dC_{\mathrm{at}}}{dt} = \epsilon(t)(1-x) - P + R_{\mathrm{veg}} + R_{\mathrm{so}} - F_{\mathrm{oc}}, \tag{2}$$
$$\frac{dC_{\mathrm{oc}}}{dt} = F_{\mathrm{oc}}, \tag{3}$$
$$\frac{dC_{\mathrm{veg}}}{dt} = P - R_{\mathrm{veg}} - L, \tag{4}$$
$$\frac{dC_{\mathrm{so}}}{dt} = L - R_{\mathrm{so}}, \tag{5}$$
$$c\frac{dT}{dt} = (F_d - \sigma T^4)a_E. \tag{6}$$

Climate variables are expressed as deviations from pre-industrial levels. Definitions of state variables and climate ‘processes’ are given in S1 Table, and baseline parameter values are provided in S2 Table. Functional forms for each process are outlined below.

Photosynthesis

Carbon uptake from the atmosphere via photosynthesis takes the following form

$$P(C_{\mathrm{at}}, T) = k_p C_{\mathrm{ve0}} k_{MM}\left(\frac{\mathrm{pCO}_{2a} - k_c}{K_M + \mathrm{pCO}_{2a} - k_c}\right)\left(\frac{(15+T)^2(25-T)}{5625}\right) \tag{7}$$

for $\mathrm{pCO}_{2a} \geq k_c$ and $-15 \leq T \leq 25$, and zero otherwise. The mixing ratio of CO_2 in the atmosphere, pCO_{2a} is defined as the ratio of moles of CO_2 in the atmosphere to the total number of moles of molecules in the atmosphere k_a. Thus

$$\mathrm{pCO}_{2a} = \frac{f_{\mathrm{gtm}}(C_{\mathrm{at}} + C_{\mathrm{at0}})}{k_a} \tag{8}$$

where $f_{\mathrm{gtm}} = 8.3259 \times 10^{13}$ is the conversion factor from gTC to moles of carbon and C_{at0} is initial level of CO_2 in the atmosphere. Note photosynthesis satisfies Michaelis-Menton kinetics in pCO_{2a} resembling increasing but saturating rates of photosynthesis as carbon in the atmosphere increases. The temperature term captures optimal photosynthesis at $T = 2$ (atmospheric temp. of 27°C) with declining rates for further increases in temperature.

Respiration

Plant respiration takes the form

$$R_{\mathrm{veg}}(T, C_{\mathrm{veg}}) = k_r C_{\mathrm{veg}} k_A e^{-\frac{E_a}{R(T+T_0)}} \tag{9}$$

which increases with the amount of carbon present in the vegetation, and also with the temperature. This provides a positive feedback with increasing carbon levels. Soil respiration takes an analogous form:

$$R_{\mathrm{so}}(T, C_{\mathrm{so}}) = k_{sr} C_{\mathrm{so}} k_B e^{-\frac{308.56}{T+T_0-227.13}}. \tag{10}$$

Turnover

There is an assumed constant fraction of plants dying in a given unit of time:

$$L(C_{\mathrm{veg}}) = k_t C_{\mathrm{veg}} \tag{11}$$

The stored carbon is then fed into the soil reservoir.

Ocean flux

Flux of CO_2 from the atmosphere to the ocean takes the form

$$F_{\mathrm{oc}}(C_{\mathrm{at}}, C_{\mathrm{oc}}) = F_0 \chi \left(C_{\mathrm{at}} - \zeta \frac{C_{\mathrm{at0}}}{C_{\mathrm{oc0}}} C_{\mathrm{oc}} \right) \tag{12}$$

where χ is characteristic solubility of CO_2 in water and ζ is the evasion factor [6]. More complex ocean models couple these parameters to chemical dynamics within the ocean itself [4], however we find good agreement when comparing this simplified climate model with the full Earth system model presented in [4].

Atmospheric Dynamics

We use the grey-atmosphere approximation as used in [4] to model atmospheric dynamics. This framework captures changes in global average surface temperature due to changes in albedo, solar flux, and the opacity of CO_2, H_2O_v and CH_4. The net downward flux of radiation absorbed at the planet's surface is given by

$$F_d = \frac{(1-A)S}{4} \left(1 + \frac{3}{4}\tau \right), \tag{13}$$

where A is the surface albedo, S is the incoming solar flux and τ vertical opacity of the greenhouse atmosphere. Expressions for each opacity are given by

$$\tau(\mathrm{CO_2}) = 1.73(\mathrm{pCO_2})^{0.263} \tag{14}$$

$$\tau(\mathrm{H_2O}) = 0.0126 \left(H P_0 e^{-(L/RT)} \right)^{0.503} \tag{15}$$

$$\tau(\mathrm{CH_4}) = 0.0231 \tag{16}$$

where pCO_2 is the mixing ratio of CO_2 in the atmosphere as defined earlier, H is the relative humidity, P_0 is the water vapor saturation constant, L is the latent heat per mole of water, and R is the molar gas constant.

2 Parameter selection

2.1 Climate parameters

All climate parameters are borrowed from the Earth System model [4] except for those governing carbon transfer with the ocean, where we use a simplified framework [6]. We calibrate these ocean parameters to recover dynamics of the full Earth System model. The relative humidity H is calibrated to give a pre-industrial temperature of 288.15K. Parameter values along with upper and lower bounds are listed in S2 Table.

2.2 Social Parameters

For reference, social dynamics are modeled by

$$\frac{dx}{dt} = \kappa x(1-x)(-\beta + f(T_f) + \delta(2x-1)). \tag{17}$$

Reasoning for baseline parameters are provided below. The effect of varying key social parameters is detailed in the manuscript.

Social learning rate (κ) determines the rate at which an alternate strategy propagates throughout a population, once the alternate strategy has a higher overall utility. Population level change in consensus can take decades (e.g. smoking as a health hazard) and so we select a value of κ accordingly. The time taken for consensus change is $O(1/\kappa)$ and so we use a baseline value of $\kappa = 0.05$ to correspond to change over a 20-year period. This is similar to previous studies modeling of evolving population consensus in socio-ecological systems [2,3]. Upper/lower bounds of κ are taken to correspond population level consensus change from 5 / 50 years respectively.

Net cost of mitigation (β) is set to one without loss of generality. Other payoff parameters are normalised to be relative to the net cost of mitigation.

Strength of social norms (δ) is a measure of the 'cost' that an individual incurs when acting against the majority opinion. In a population with complete consensus ($x = 1, 0$), the cost to adopt the alternate strategy is exactly δ. We assume this cost is on the same order as the net cost of mitigative practices (β) and so we investigate δ for the range $(0.5, 1.5)$.

Cost of warming ($f(T_f)$) represents the perceived costs associated with a global temperature anomaly of T_f degrees Celsius. Despite the growing acceptance of climate change and its consequences, the rate of global CO_2 emissions per capita shows little sign of slowing down (S8 Fig). Temperature records report a current temperature anomaly of 1°C above pre-industrial values and CO_2 emissions per capita are hovering at approximately $1.3\ \mathrm{tC\,yr^{-1}}$, the highest rate in history. When modeling an individual's perceived cost of global warming at a temperature of T, we therefore use a sigmoidal function (Methods). This assumes small temperature anomalies (on the order of 1°C) receive low response, as is observed in empirical data (S8 Fig). At a specified threshold temperature, the response function increases in a non-linear fashion, capturing the expected non-linear increase in frequency of climate change disasters as temperature increases [5]. The cost function eventually saturates for high enough temperature.

References

[1] Dirk Helbing. *Quantitative sociodynamics: stochastic methods and models of social interaction processes*. Springer Science & Business Media, 2010.

[2] Kirsten A Henderson, Chris T Bauch, and Madhur Anand. Alternative stable states and the sustainability of forests, grasslands, and agriculture. *Proceedings of the National Academy of Sciences*, 113(51):14552–14559, 2016.

[3] Clinton Innes, Madhur Anand, and Chris T Bauch. The impact of human-environment interactions on the stability of forest-grassland mosaic ecosystems. *Scientific reports*, 3:2689, 2013.

[4] Timothy M Lenton. Land and ocean carbon cycle feedback effects on global warming in a simple earth system model. *Tellus B: Chemical and Physical Meteorology*, 52(5):1159–1188, 2000.

[5] Timothy M Lenton, Hermann Held, Elmar Kriegler, Jim W Hall, Wolfgang Lucht, Stefan Rahmstorf, and Hans Joachim Schellnhuber. Tipping elements in the earth's climate system. *Proceedings of the national Academy of Sciences*, 105(6):1786–1793, 2008.

[6] KE Muryshev, AV Eliseev, II Mokhov, and AV Timazhev. A lag between temperature and atmospheric co2 concentration based on a simple coupled model of climate and the carbon cycle. In *Doklady Earth Sciences*, volume 463, pages 863–867. Springer, 2015.
