# Supplementary material for: Charting pathways to climate change mitigation in a coupled socio-climate model
Source: PLoS Comput Biol. 2019 Jun 6;15(6):e1007000. doi: 10.1371/journal.pcbi.1007000 (PMC6553685; doi:10.1371/journal.pcbi.1007000)
Supplement: S1 Table — (PDF) [file pcbi.1007000.s002.pdf]

Variable / Process	Definition	Unit
x	proportion of mitigators in population	1
C_{at}	deviation of atmospheric CO2 (from pre-industrial 1800)	GtC
C_{oc}	deviation of CO2 in ocean	GtC
C_{veg}	deviation in CO2 in vegetation	GtC
C_{so}	deviation in CO2 in soil	GtC
T	deviation in temperature	K
$\epsilon(t)$	CO_2 emissions in absence of mitigation	GtC/yr
P	carbon uptake from photosynthesis	GtC/yr
R_{veg}	respiration from vegetation	GtC/yr
R_{so}	respiration from soil	GtC/yr
F_{oc}	flux of CO2 from atmosphere to ocean	GtC/yr

Table 1: **Labels for state variables and dynamic processes in the socio-climate model.**
