# Supplementary material for: Charting pathways to climate change mitigation in a coupled socio-climate model
Source: PLoS Comput Biol. 2019 Jun 6;15(6):e1007000. doi: 10.1371/journal.pcbi.1007000 (PMC6553685; doi:10.1371/journal.pcbi.1007000)
Supplement: S2 Table — (PDF) [file pcbi.1007000.s003.pdf]

Parameter	Definition	Baseline values / intervals	Unit	Source
C_{at0}	initial CO_2 in atmosphere	$(590, 596, 602)$	GtC	[4, 6]
C_{ao0}	initial CO_2 in ocean reservoir	$(1.4, 1.5, 1.6) \times 10^5$	GtC	[7]
C_{veg0}	initial CO_2 in vegetation reservoir	$(540, 550, 560)$	GtC	[4, 6]
C_{veg0}	initial CO_2 in soil reservoir	$(1480, 1500, 1520)$	GtC	[4, 6]
T_0	initial average atmospheric temperature	$(288, 288.15, 288.3)$	K	[6]
k_p	photosynthesis rate constant	$(0.175, 0.184, 0.193)$	yr^{-1}	[4, 6]
k_{MM}	photosynthesis normalising constant	1.478	1	[6]
k_c	photosynthesis compensation point	$(26, 29, 32) \times 10^{-6}$	1	[3, 6]
K_M	half-saturation point for photosynthesis	$(108, 120, 132) \times 10^{-6}$	1	[6]
k_a	mole volume of atmosphere	1.773×10^{20}	moles	[6, 9]
k_r	plant respiration constant	$(0.0828, 0.092, 0.1012)$	yr^{-1}	[4, 6]
k_A	plant respiration normalising constant	8.7039×10^9	1	[6]
E_a	plant respiration activation energy	$(54.63, 54.83, 55.03)$	J mol^{-1}	[2, 6]
k_{sr}	soil respiration rate constant	$(0.0303, 0.034, 0.037)$	yr^{-1}	[4, 6]
k_B	soil respiration normalising constant	157.072	1	[6]
k_t	turnover rate constant	$(0.0828, 0.092, 0.1012)$	yr^{-1}	[4, 6]
c	specific heat capacity of Earth's surface	$(4.22, 4.69, 5.16) \times 10^{23}$	JK^{-1}	[4, 6]
a_E	Earth's surface area	5.101×10^{14}	m^2	universal
σ	Stefan-Boltzman constant	5.67×10^{-8}	$\text{Wm}^{-2}\text{K}^{-4}$	universal
L	latent heat per mole of water	43 655	mol^{-1}	universal
R	molar gas constant	8.314	$\text{J mol}^{-1}\text{ K}^{-1}$	universal
H	relative humidity	0.5915	1	calibrated
A	surface albedo	$(0.203, 0.225, 0.248)$	yr^{-1}	[5, 6]
S	solar flux	$(1231, 1368, 1504)$	Wm^{-2}	[5, 6]
$\tau(CH_4)$	methane opacity	$(0.0208, 0.0231, 0.0254)$	1	[1, 6]
P_0	water vapor saturation constant	$(1.26, 1.4, 1.54) \times 10^{11}$	Pa	[6, 8]
F_0	ocean flux rate constant	$(2.25, 2.5, 2.75) \times 10^{-2}$	yr^{-1}	[7]
χ	characteristic CO_2 solubility	$(0.2, 0.3, 0.4)$	1	calibrated
ζ	evasion factor	$(40, 50, 60)$	1	calibrated
κ	social learning rate	$(0.02, 0.05, 0.2)$	yr^{-1}	-
β	net cost of mitigation	$(0.5, 1, 1.5)$	1	-
δ	strength of social norms	$(0.5, 1, 1.5)$	1	-
$f_{\max}$	maximum of warming cost function $f(T)$	(4,5,6)	1	-
ω	nonlinearity of warming cost function $f(T)$	$(1, 3, 5)$	K^{-1}	-
T_c	critical temperature of $f(T)$	$(2.4, 2.5, 2.6)$	K	-
t_p	# previous years used for temperature projection	10	yr	-
t_f	# years ahead for temperature projection	$(0, 25, 50)$	yr	-
s	half-saturation time for $\epsilon(t)$ from 2014	$(30, 50, 70)$	yr	-
$\epsilon_{\max}$	maximum change in $\epsilon(t)$ from 2014	$(4.2, 7, 9.8)$	GtC yr^{-1}	-
x_0	initial proportion of mitigators	$(0.01, 0.05, 0.1)$	1	-

Table 1: **Definitions and values for the parameters in the socio-climate model.** Parameters values given as a tuple provide the lower bound, baseline, and upper bound values respectively.

References

[1] Jérôme Chappellaz, JM Barnola, D Raynaud, Ye S Korotkevich, and C Lorius. Ice-core record of atmospheric methane over the past 160,000 years. *Nature*, 345(6271):127, 1990.

[2] AD Friend, AK Stevens, RG Knox, and MGR Cannell. A process-based, terrestrial biosphere model of ecosystem dynamics (hybrid v3. 0). *Ecological Modelling*, 95(2-3):249–287, 1997.

[3] Roger M Gifford. Implications of co 2 effects on vegetation for the global carbon budget. In *The global carbon cycle*, pages 159–199. Springer, 1993.

[4] John T Houghton. *Climate change 1995: The science of climate change: contribution of working group I to the second assessment report of the Intergovernmental Panel on Climate Change*, volume 2. Cambridge University Press, 1996.

[5] James F Kasting, Daniel P Whitmire, and Ray T Reynolds. Habitable zones around main sequence stars. *Icarus*, 101(1):108–128, 1993.

[6] Timothy M Lenton. Land and ocean carbon cycle feedback effects on global warming in a simple earth system model. *Tellus B: Chemical and Physical Meteorology*, 52(5):1159–1188, 2000.

[7] KE Muryshev, AV Eliseev, II Mokhov, and AV Timazhev. A lag between temperature and atmospheric co2 concentration based on a simple coupled model of climate and the carbon cycle. In *Doklady Earth Sciences*, volume 463, pages 863–867. Springer, 2015.

[8] Shinichi Nakajima, Yoshi-Yuki Hayashi, and Yutaka Abe. A study on the "runaway greenhouse effect" with a one-dimensional radiative–convective equilibrium model. *Journal of the Atmospheric Sciences*, 49(23):2256–2266, 1992.

[9] JR Toggweiler and Jorge L Sarmiento. Glacial to interglacial changes in atmospheric carbon dioxide: The critical role of ocean surface water in high latitudes. *The carbon cycle and atmospheric CO2: natural variations archean to present*, 32:163–184, 1985.
