# Supplementary figures and images for: Charting pathways to climate change mitigation in a coupled socio-climate model
Source: PLoS Comput Biol. 2019 Jun 6;15(6):e1007000. doi: 10.1371/journal.pcbi.1007000 (PMC6553685; doi:10.1371/journal.pcbi.1007000)

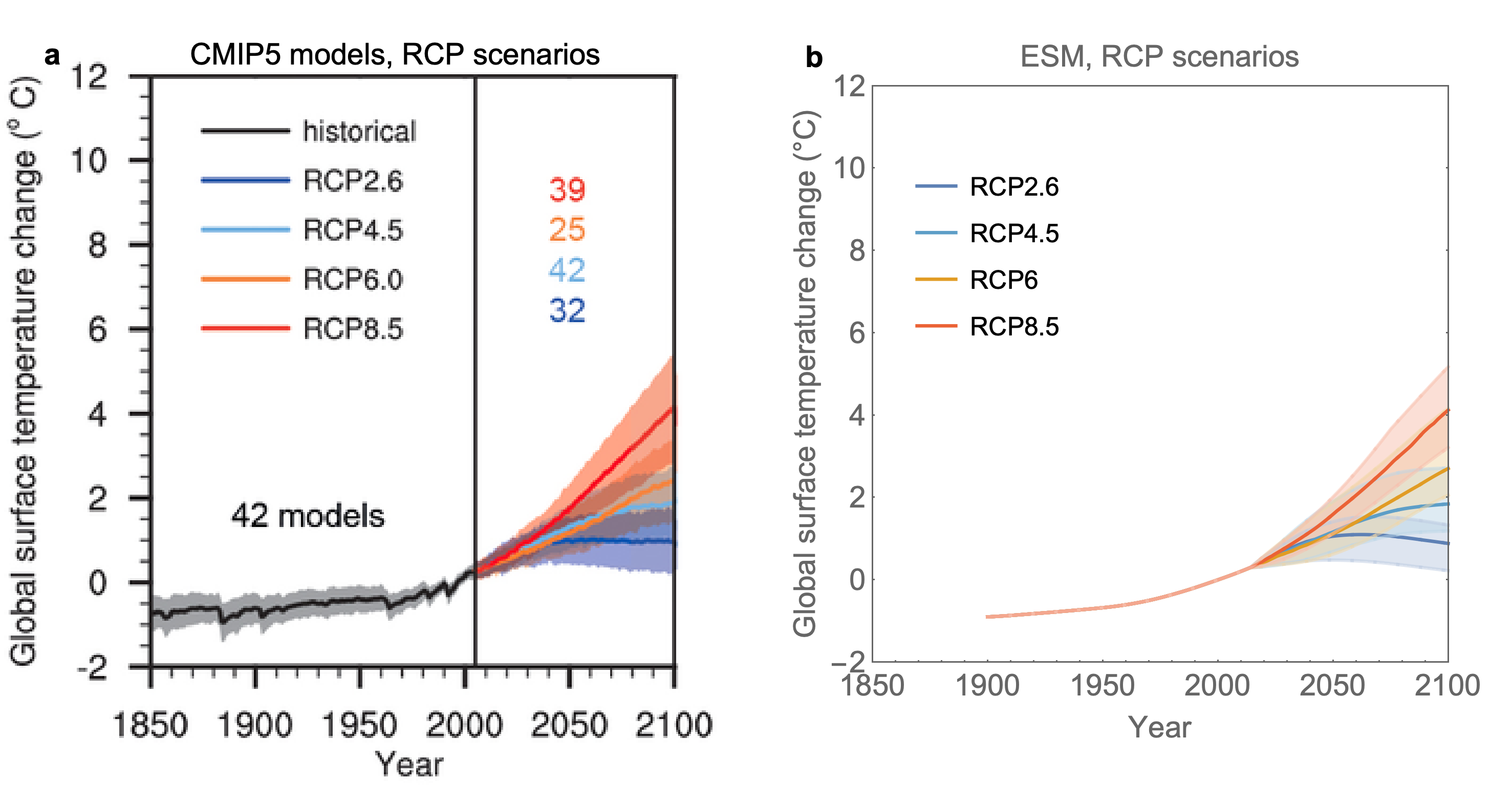

Supplement: S1 Fig — a. Ensemble of simulations from the Coupled Model Intercomparison Project Phase 5 (CMIP5) using the Representative Concentration Pathways (RCPs) as emission scenarios (figure from the IPCC Fifth Assessment Report [45]). Displayed are 95% confidence intervals based on annual means. Numbers and their colour denote the number of models used for each RCP scenario. b. Ensemble of simulations from the simple Earth system model that we use in our socio-climate model under the same RCP emission scenarios. Parameters are drawn from triangular distributions with upper and lower bounds given in S2 Table. (TIFF) [file pcbi.1007000.s004.tiff]

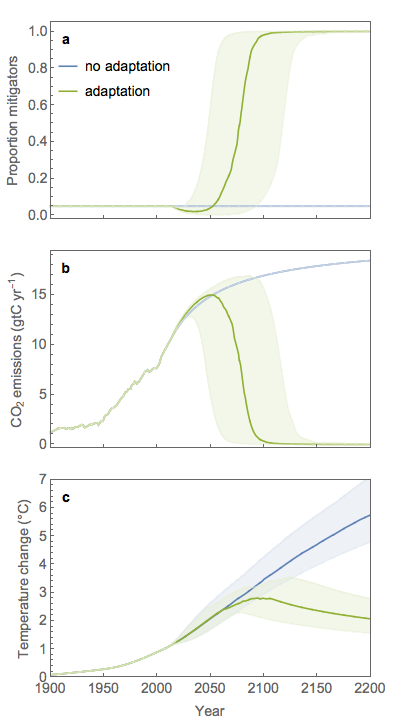

Supplement: S2 Fig — Removing adaptive behaviour from the model (by forcing the proportion of mitigators to remain at a constant, low value) results in saturating emissions and temperature increasing indefinitely (at least over the next two centuries). This is akin to the RCP8.5 scenario in the latest IPCC report (trajectory shown in Fig 1). Simulations above use parameter values drawn from triangular distributions with upper and lower bounds given in S2 Table. (TIFF) [file pcbi.1007000.s005.tiff]

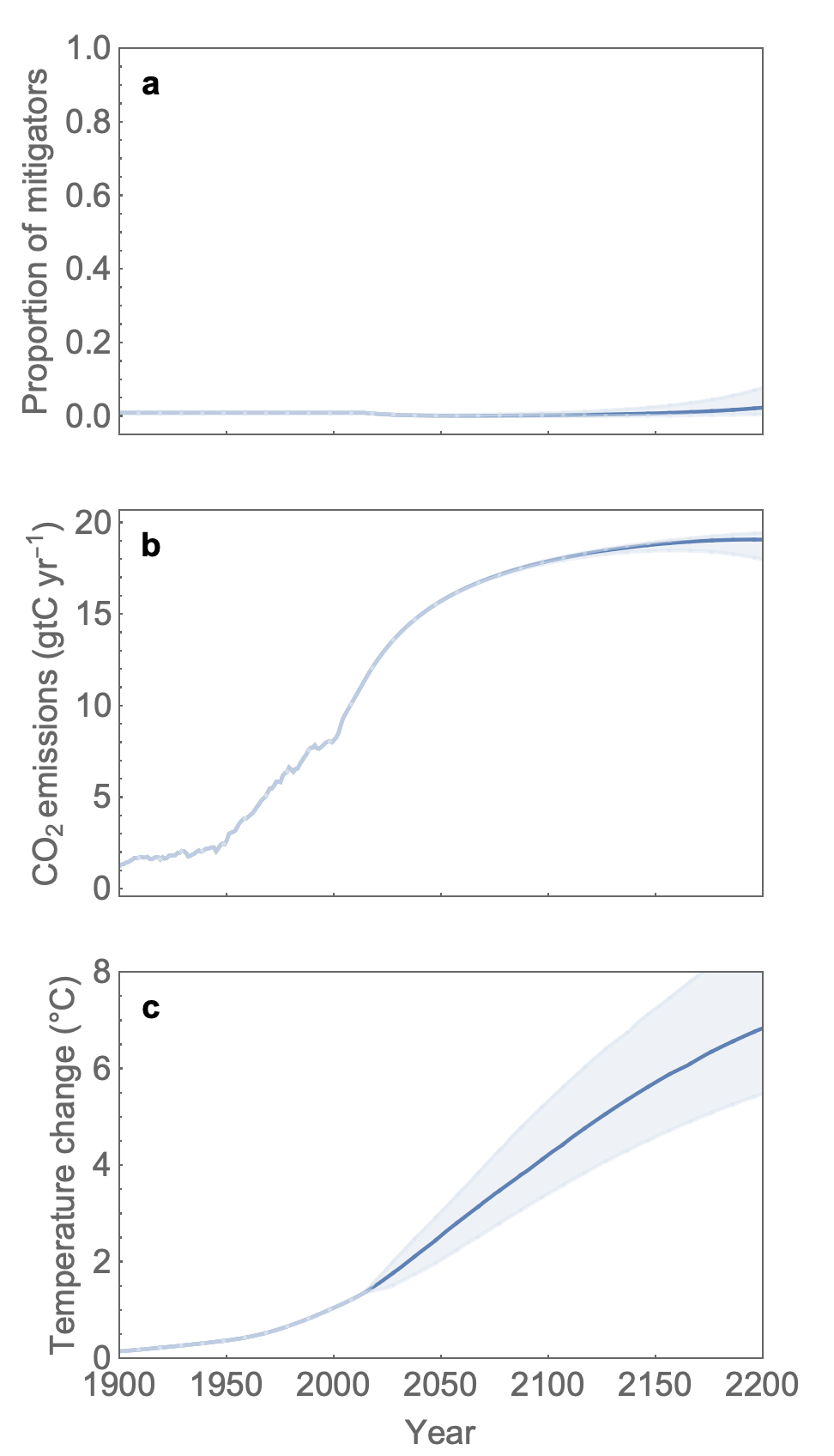

Supplement: S3 Fig — Setting the social parameters to their bound that most favour non-mitigative behaviour, we get no spread of mitigative behaviour within the considered time-frame. This causes the temperature to increase in a manner similar to the RCP 8.5 scenario (S1 Fig). Fixed parameter values are κ = 0.02, β = 1.5, δ = 1.5, fmax = 4, x0 = 0.01. All other parameter values are drawn from triangular distributions with upper and lower bounds given in S2 Table. (TIFF) [file pcbi.1007000.s006.tiff]

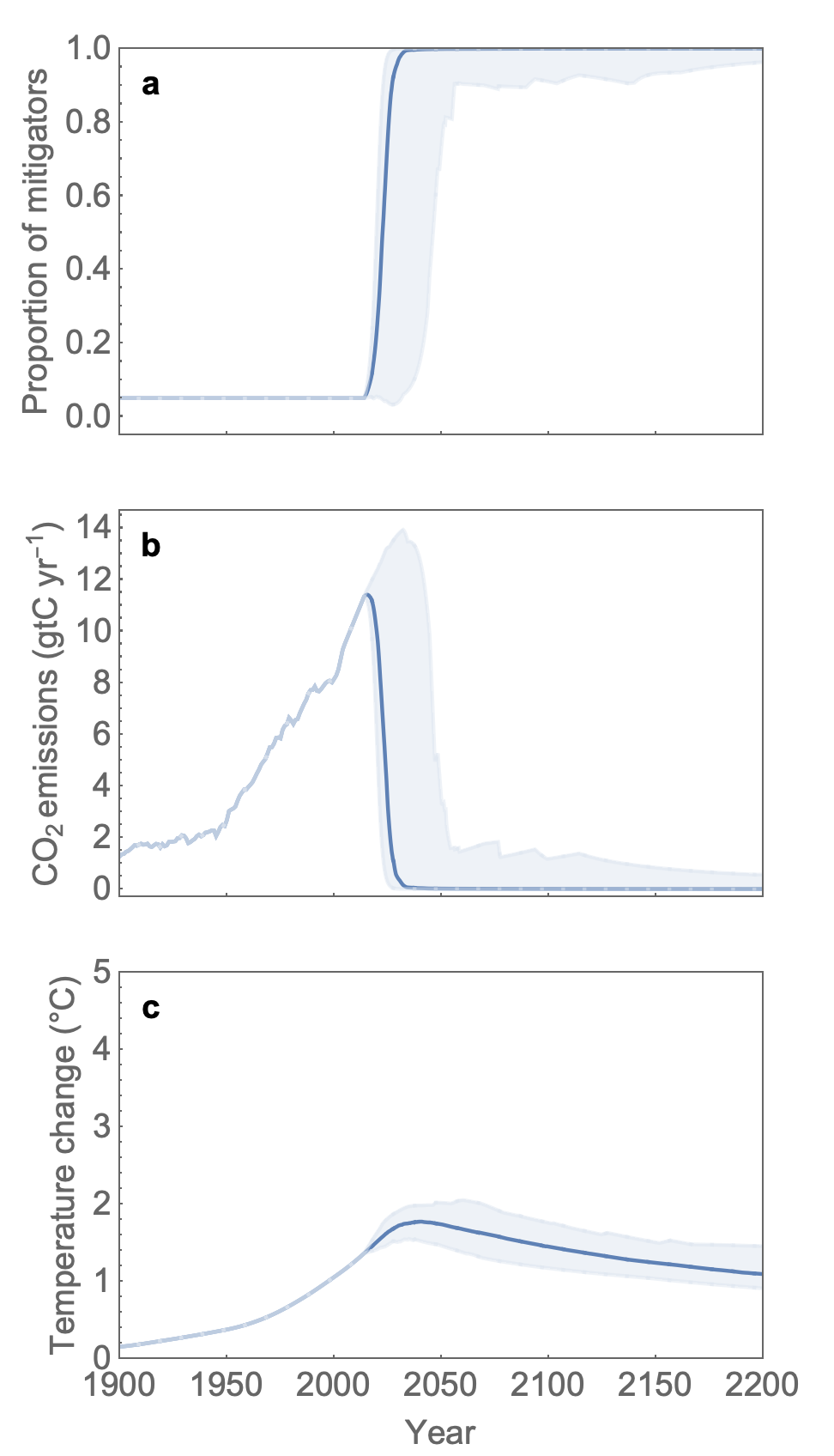

Supplement: S4 Fig — Setting the social parameters to their bound that least favour non-mitigative behaviour, we get very early spread of mitigative behaviour. This causes the temperature to evolve in a manner most similar to the RCP 2.6 scenario where temperature change stays below 2 degrees Celsius (S1 Fig). Fixed parameter values are κ = 0.2, β = 0.5, δ = 0.5, fmax = 6, tf = 50. All other parameter values are drawn from triangular distributions with upper and lower bounds given in S2 Table. (TIFF) [file pcbi.1007000.s007.tiff]

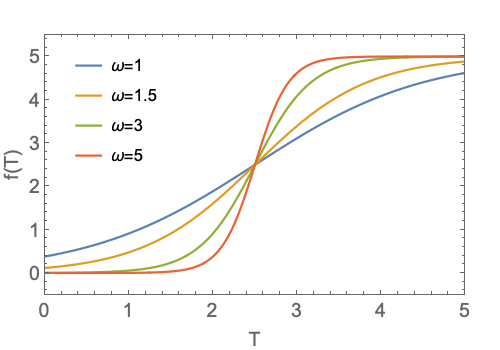

Supplement: S5 Fig — The incentive of individuals to mitigate is in part based on their perceived costs of climate change f at some projected temperature T. We adopt a sigmoidal response curve for f(T) with variable curvature ω and horizontal shift Tc (the explicit form of f(T) is provided in Methods). This form captures the expected non-linear increase in climate change impacts (cost) as temperature increases. (TIFF) [file pcbi.1007000.s008.tiff]

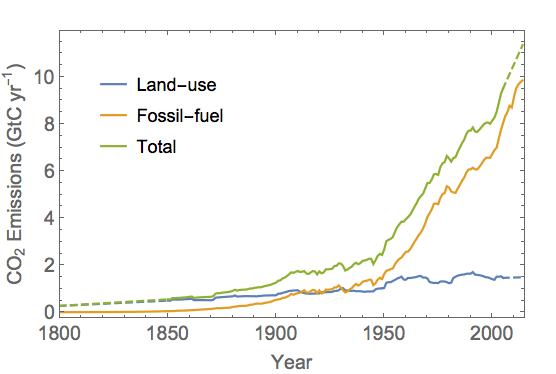

Supplement: S6 Fig — Data from the CDIAC on carbon emissions due to fossil-fuel burning and land-use changes, during the years 1800-2014. Land-use data is only available from 1850-2005 and so we linearly extrapolate (dashed line) to match the range of the fossil-fuel data. The sum of the emission trajectories is used to drive the model up to the year 2014, from which point the behavioural component of the socio-climate model is initiated. (TIFF) [file pcbi.1007000.s009.tiff]

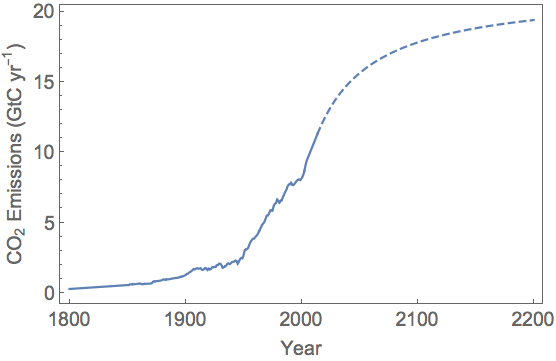

Supplement: S7 Fig — In the socio-climate model, the factor ϵ(t) corresponds to the global CO2 emissions should there be no change in human behaviour. Preceding 2014, ϵ(t) takes the historical emission trajectory (solid line) as human behaviour is not modelled here. Post 2014, ϵ(t) follows a saturating function (dashed line) to capture the saturation of global population size and energy needs. Details of this functional form can be found in the Methods section. (TIFF) [file pcbi.1007000.s010.tiff]

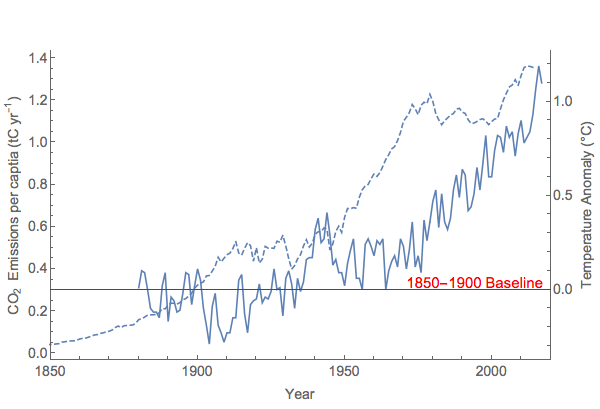

Supplement: S8 Fig — Temperature anomaly (solid line) above the 1850-1900 baseline value and CO2 emissions per capita (dashed line) are shown for the 1850-2014. Data was obtained from the CDIAC data repository [35]. It is clear that despite exceeding a 1 degree temperature anomaly, the global emissions per capita of CO2 show no obvious signs of decreasing. The current temperature anomaly is not yet high enough to spark a global decrease in emissions. (TIFF) [file pcbi.1007000.s011.tiff]

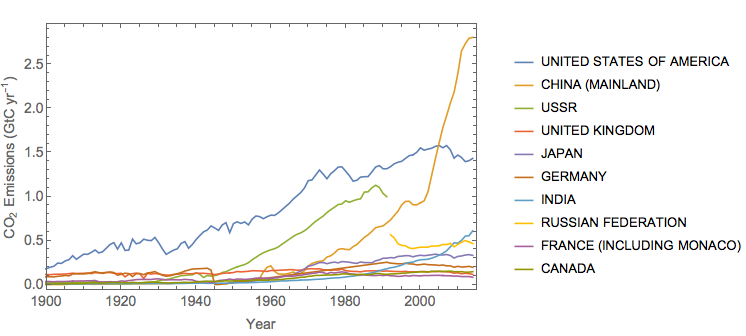

Supplement: S9 Fig — Total industrial CO2 emissions from the ten countries with the greatest cumulative output. Data was obtained from the CDIAC data repository [35]. Note the termination of the green curve marks the dissolution of the Soviet Union and the beginning of the yellow curve marks the formation of the Russian Federation. (TIFF) [file pcbi.1007000.s012.tiff]

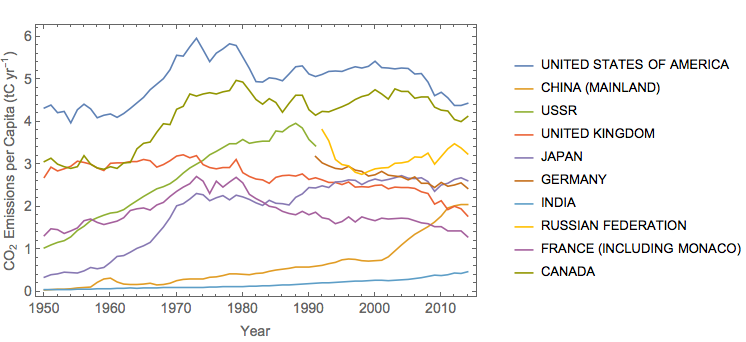

Supplement: S10 Fig — Total industrial CO2 emissions per capita from the ten countries with the greatest cumulative output. Data was obtained from the CDIAC data repository [35]. From 1950-1980 we observe strong increases in emissions per capita among most of these countries as is seen globally in S8 Fig. Many of the countries peak in 1980 and follow slight downwards trends demonstrating a degree of country-level movement towards mitigation. Globally, this is not the case, however. (TIFF) [file pcbi.1007000.s013.tiff]
